# Medium-chain and long-chain fatty acids are associated with diarrheal predominant irritable bowel syndrome revealed by DESI-MSI

**DOI:** 10.1007/s00535-023-02030-6

**Published:** 2023-08-14

**Authors:** Yanli Zhang, Huiting Zhu, Shiyu Du, Huifen Wang, Hui Li, Miao Wang, Bing Shao

**Affiliations:** 1 Department of Gastroenterology, China-Japan Friendship Hospital, Beijing, 100029 China; 2 Beijing Key Laboratory of Diagnostic and Traceability Technologies for Food Poisoning, Beijing Centers for Disease Control and Preventative Medical Research, Beijing, 100013 China; 3 Department of Gastroenterology, First Hospital of Qinhuangdao, Qinhuangdao, 066000 Hebei China

**Keywords:** Irritable bowel syndrome, Metabonomics, DESI-MSI, Fatty acids

## Abstract

**Background:**

Irritable bowel syndrome (IBS) is one of the most common functional bowel disorders, but its pathogenesis remains unknown. Its development may be linked to intestinal dysmetabolism, directly and indirectly. The present study aimed to screen the differentially expressed small molecular substances in the mucosa of the colon between IBS with diarrhea (IBS-D) patients and healthy subjects and explore the pathogenesis of IBS-D.

**Methods:**

In this pilot study, the metabolites of colonic mucosa in ten patients with IBS-D and six healthy controls (HC) were analyzed by DESI-MSI. We also mapped the spatial distribution of the screened differential metabolites from samples of the IBS-D group and HC group.

**Results:**

The results showed that 20 metabolites in the colonic mucosa of IBS-D were significantly more abundant, while the other 77 substances were significantly reduced. Enrichment analysis of 97 differential metabolites and KEGG pathway analysis revealed that six medium-chain and long-chain fatty acids were determined to be most overrepresented in the IBS-D group compared to the HC group. Four of these six fatty acids are all PUFAs. The DESI–MSI results suggested that these fatty acids were localized in the colonic mucosa and confirmed the differences in these fatty acids between IBS-D and HC.

**Conclusions:**

Medium-chain and long-chain fatty acids localized in the colonic mucosa are likely to be potential indicators for the differentiation of IBS-D from healthy subjects which may have implications in the mechanisms and possible preventive measures against IBS.

**Clinical trial registry registration number:**

ChiCTR2200060224.

**Supplementary Information:**

The online version contains supplementary material available at 10.1007/s00535-023-02030-6.

## Introduction

Irritable bowel syndrome (IBS) is a common functional bowel disorder. To date, IBS is diagnosed based on symptoms criteria with no evidence of colonic histological structural, biochemical and pathological abnormalities [1]. The precise aetiology and pathophysiology of IBS are incompletely understood, despite extensive interest and investigation. The pathophysiologic mechanisms of IBS include but are not limited to, altered gastrointestinal motility, visceral hyperalgesia, altered microbiota metabolism, immune activation, and low-grade inflammation [2, 3].

The recent metabonomics technique examines small molecules in biological samples and detects subtle changes reflecting different physiological and pathological conditions, which has also been used to identify potential metabolic biomarkers for IBS. Alterations in metabolite production may be related to the manifestation of IBS symptoms. The propionic acid and butyric acid are increased in IBS with diarrhea (IBS-D) patients in serum but not in feces by gas chromatography–mass spectrometric (GC–MS) method [4]. Another feces analysis study using GC–MS indicated there were 14 differential metabolites in the murine IBS model group compared with the controls, including amino acids, short-chain fatty acids (SCFAs) and steroid hormones [5]. However, most of the results on metabolism characteristics of IBS came from feces, urine and blood samples. In fact, we don’t know what is exactly happening to the colonic mucosal metabolism, which must be very helpful in understanding of IBS.

Although the global metabonomics technique is a powerful method for discovering biomarkers, spatial distribution information of metabolites is unavailable. Desorption electrospray ionization-mass spectrometry imaging (DESI-MSI) as a new technology, can map metabolites to tissue in situ and be co-registered with corresponding pathological structures [6]. The advantages of DESI-MSI are the absence of matrix deposition and the non-destructive nature of the analysis, which means that tissue sections after DESI-MSI scanning can directly undergo subsequent histologic staining [7–9]. DESI-MSI has been initially applied in studies on malignant tumors [6, 10, 11].

In this study, we used DESI-MSI to compare the metabolic differences and spatial distribution of small molecules in the colonic mucosa between IBS-D patients and healthy controls. We aim to elucidate the small molecular metabolites that may be involved in the pathogenesis of IBS-D, analyze the distribution characteristics of small molecular substances in IBS-D colonic mucosa and explore the possible investigation of mucosa biomarkers of IBS-D.

## Materials and methods

### Chemicals and reagents

Carboxymethyl cellulose was purchased from Hualan Chemical Co. Ltd (Shanghai, China). LC–MS grade methanol was purchased from Thermo Fisher Scientific (Pittsburgh, PA, USA).

### Patients and study design

We performed a prospective study in patients with IBS-D and healthy controls (HC). Ten adult IBS-D patients meeting the Rome IV criteria were collected from the Department of Gastroenterology in our hospital between January 2022 and May 2022. Disease severity of IBS-D was measured by the IBS Symptom Severity Scale (IBS-SSS), which includes five items (severity and frequency of abdominal pain, abdominal distension, bowel habit dissatisfaction and life interference) [12]. Scores for the five items were summed to achieve a total score ranging from 0 to 500. IBS severity was then classified as mild (75–174), moderate (175–299) or severe (300–500). The healthy subjects were the volunteers from communities. Six healthy subjects without organic intestinal diseases or gastrointestinal symptoms were recruited as controls.

Inclusive criteria for all subjects were: an age between 18 and 65 years; normal blood count; serum creatinine, alanine aminotransferase (ALT) and alkaline phosphatase (ALP) within reference values; normal thyroid function. We excluded participants for the following reasons: history of major gastrointestinal surgery; malignant tumours; history of polyp in 3 years; diarrhoea due to other medical illnesses (e.g., diabetes mellitus, inflammatory bowel disease, hyperthyroid, neurological diseases); pregnancy and breastfeeding females; chronic opioids or anti-depressants use. Participants were not allowed to take any antibiotics, probiotics, prebiotics or antidiarrhoea within 4 weeks before recruitment. Meanwhile, all subjects were not allowed to drink alcohol and coffee, as well as taking nutritional supplements such as vitamins and dietary fiber for 2 weeks before inclusion in the experiments.

All IBS-D patients and healthy subjects underwent colonoscopy, and 1 biopsy specimen was taken from the ileocecal junction by biopsy forceps, which was used for DESI-MSI analysis and Haematoxylin and eosin (H&E) staining (Supplementary Fig. 1).

The study protocol was approved by the Human Ethical Committee of China-Japan Friendship Hospital, and all participants signed informed consent.

### Sample preparation

The ileocecal specimens were snap-frozen in liquid nitrogen immediately after biopsy removal and stored at – 80 °C until further processing. The frozen samples were embedded in 5% sodium carboxymethyl cellulose (CMC). Frozen sections (20 μm thick) were serially cut using a cryotome, mounted on glass slides, and stored at – 80 °C for DESI–MSI, while adjacent 9 μm-thick frozen sections were mounted on glass slides and stained with H&E.

### DESI-MSI

All MSI experiments were performed using the SYNAPT G2-Si HDMS DESI XS instrument (Waters, Milford, MA, USA) and a pump of Harvard Apparatus Pump11 Elite. Glass slides containing 20 μm slices were subjected to DESI–MSI in the positive and negative ion modes over the mass range *m*/*z* 100–1200. The 1000 peaks with the highest intensities were then chosen. The spray solvent for DESI was methanol/water in a ratio of 98:2, containing 200 ppb leucine encephalin, and injected at a rate of 3 μl/min. The parameter settings were as follows: capillary temperature, 150 °C; capillary voltage, 3.5 kV; nitrogen spray, 0.45 MPa. Tissues were performed in constant velocity scan mode with a velocity of 100 μm/s and set at a spatial resolution of 50 μm to acquire DESI–MS images.

The mass spectral data were processed and 2D spatially resolved ion images were generated by the high-definition imaging (HDI) platform version 1.5 (Waters). Leucine encephalin (positive, *m*/*z* 556.2771; negative, *m*/*z* 554.2615) was used as the lock mass. The mass spectra were normalized to the total ion current.

### H&E staining and microscopic observation

The adjacent slices were stained with H&E according to the manufacturer’s protocol. Images were observed by Nikon ECLIPSE 80i microscope with [4x/0.13 Plan Fluor] objective lens. Two pathology specialists from the Department of Pathology of China-Japan Friendship Hospital were invited to read the H&E slices and all samples in IBS-D were excluded from the microscopic colitis.

### Data processing and statistical analysis

Four equal-area regions of interest (ROI) were selected on the mucosa layer of each DESI image compared with the H&E staining images. EZinfo 3.0 (Waters) was used to further process the DESI-MSI data. Multivariate analyses were applied to the metabolite data. An orthogonal partial least squares discriminant analysis (OPLS-DA) served as a prediction model to identify the potential differences between the two groups. Volcano scatter plots were applied for the identification of differentiating metabolites for which the fold change (FC) for each metabolite between IBS-D and healthy control was calculated (i.e. metabolite A in the IBS group/metabolite A in the healthy group). Significance (− log10 (*P* value < 0.05), Student’s *t* test) versus log2 (mean fold change) were plotted. According to the OPLS-DA, differential metabolites were found through the S-plot.

The acquired accurate molecular information (*m*/*z*) of metabolites was further matched and annotated in the HMDB (http://www.hmdb.ca) and LIPID MAPS database (http://www.lipidmaps.org). Subsequently, all identified metabolites were classified using the Kyoto Encyclopedia of Genes and Genomes database (KEGG) (http://www.genome.jp/kegg/). Finally, the most relevant pathways were depicted by MetaboAnalyst, which used KEGG pathway enrichment analysis and topology analysis.

Statistical analysis was performed using the dedicated statistical software SPSS (version 26.0; IBM Corp, NY, USA) and Graph Prism version 8.0. The Student *t* test was used for the comparisons between the IBS-D group and the HC group. Statistical significance was defined as *P* < 0.05.

## Results

In total, our study comprised ten patients with IBS-D and six healthy subjects. The clinical characteristics of the two groups are shown in Supplementary Table 1. There were no significant differences in age and BMI between the two groups. The mean score of IBS-SSS in patients with IBS-D was 257.7.

**Fig. 1:**
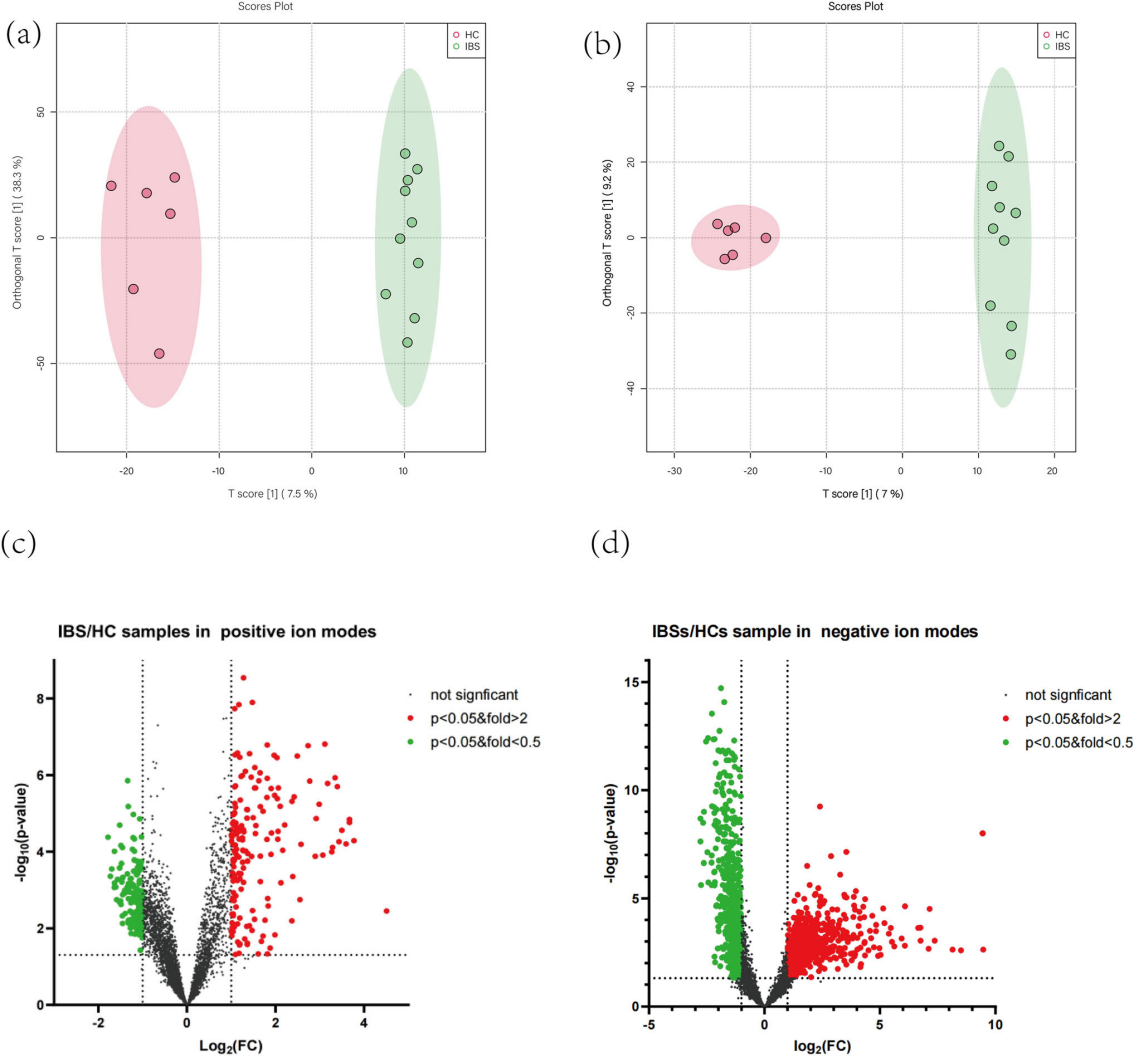
OPLS-DA plots and volcano plots of the metabolite profiles of IBS-D and HC in both positive (**a**, **c**) and negative (**b**, **d**) ion modes. The OPLS-DA score scatter plot **a** in positive ion mode and **b** in negative ion mode showed that the metabolite profiles of colonic mucosa of the IBS-D (green) and HC (red) samples differed obviously. The differential metabolites that OPLS-DA determined met the conditions of FC > 2 and *P* < 0.05 are shown in the volcano plots for both positive ion mode (**c**) and negative ion mode (**d**). Every spot represents a metabolite in the colonic mucosa. The red spots stand for the metabolites which were markedly higher detected in IBS-D than in HC, while the green spots stand for these significantly lower detected values in IBS-D. The black spots mean that there is no significant different metabolites between the two groups

DESI-MSI in combination with the OPLS-DA model was performed to compare metabolic profiles of IBS-D and HC. We obtained a total of 5053 metabolic signal peaks in the positive mode and 4270 metabolic signal peaks in the negative mode from IBS-D and HC, respectively. OPLS-DA model was applied to discriminate between the two groups (Fig. 1a, b). The OPLS-DA model was validated by a permutation test, indicating that the OPLS-DA model was not overfitting, as the test showed that the slopes of *R*^2^ and *Q*^2^ were greater than 0.5 in both the positive and negative ion modes. As shown in the OPLS-DA model (Fig. 1a, b), the metabolic profiles of the two groups differed significantly in both positive and negative ion modes. The volcano plots (Fig. 1c, d) showed the compounds that met both FC > 2 and *P* < 0.05 between the two groups in both the positive and negative ion modes. When compared with the HC group, the higher detected metabolites of colonic mucosa in IBS-D patients were represented by red spots, lower ones were represented by green spots, and black spots means no significant difference between the two groups.

**Fig. 2:**
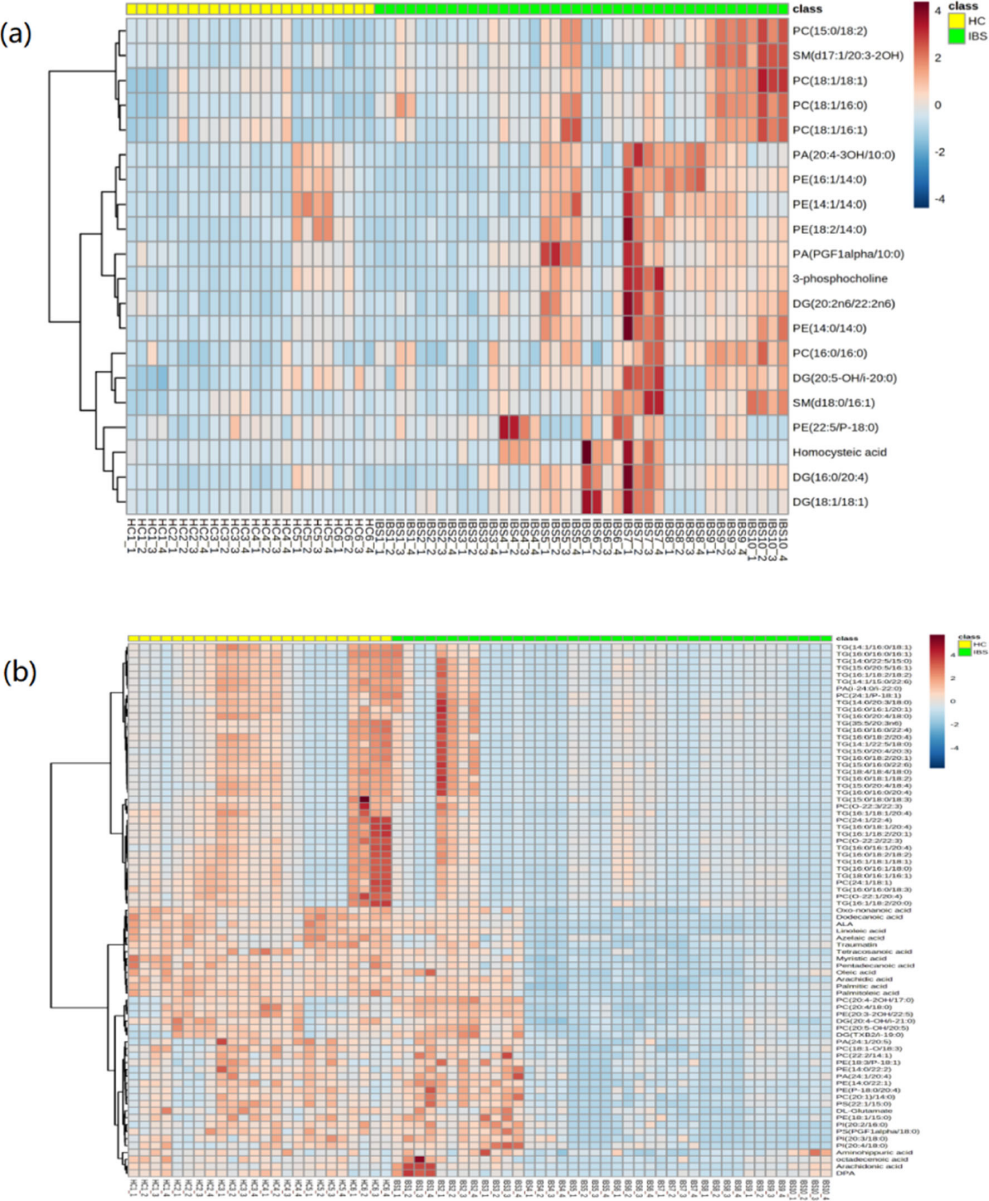
Hierarchical clustering heat map of prominent differential metabolites between the IBS-D group and HC group. The colour-coding scale shows the relative intensity of each differential metabolite: red, high intensity; blue, low intensity; white, average intensity. The horizontal axis represents the samples and the vertical axis represents the differential metabolites. Every sample has selected 4 ROIs for analysis. The metabolites in **a** were more abundant in the IBS-D group than HC group, and the metabolites were enriched in the HC group than the IBS-D group in **b**

Comparing the differences of metabolites in colonic mucosa, there were 97 significant differential metabolites between the two groups. With the exception of 2 amino acids, 95 metabolites are lipids. Of these lipids, 39 were glycerophospholipid (GLP), 17 were fatty acid (FA), 2 were sphingomyelin (SM), 37 were acylglycerol [31 triglyceride (TG) and 6 diglyceride (DG)]. Compared with the HC group, 20 substances in the mucous layer were significantly more abundant, while the other 77 substances were significantly reduced in the IBS-D group (Supplementary Table 2 and Supplementary Table 3). Hierarchical clustering heat map also illustrates patterns of changes in major metabolites between the two groups (Fig. 2a, b). It indicates 13 GLPs, 2 SMs, 4 DGs and 1 amino acid were more abundant in the IBS-D group (Fig. 2a), and the others were downregulated (Fig. 2b), including 17 FAs, 26 GLPs, 33 acylglycerols and 1 amino acid. From the perspective of lipid classification, we found that all 17 fatty acids in differential metabolites were downregulated in IBS-D patients, and they all belong to medium-chain and long-chain fatty acids (C6 and longer) (Supplementary Table 2).

**Fig. 3:**
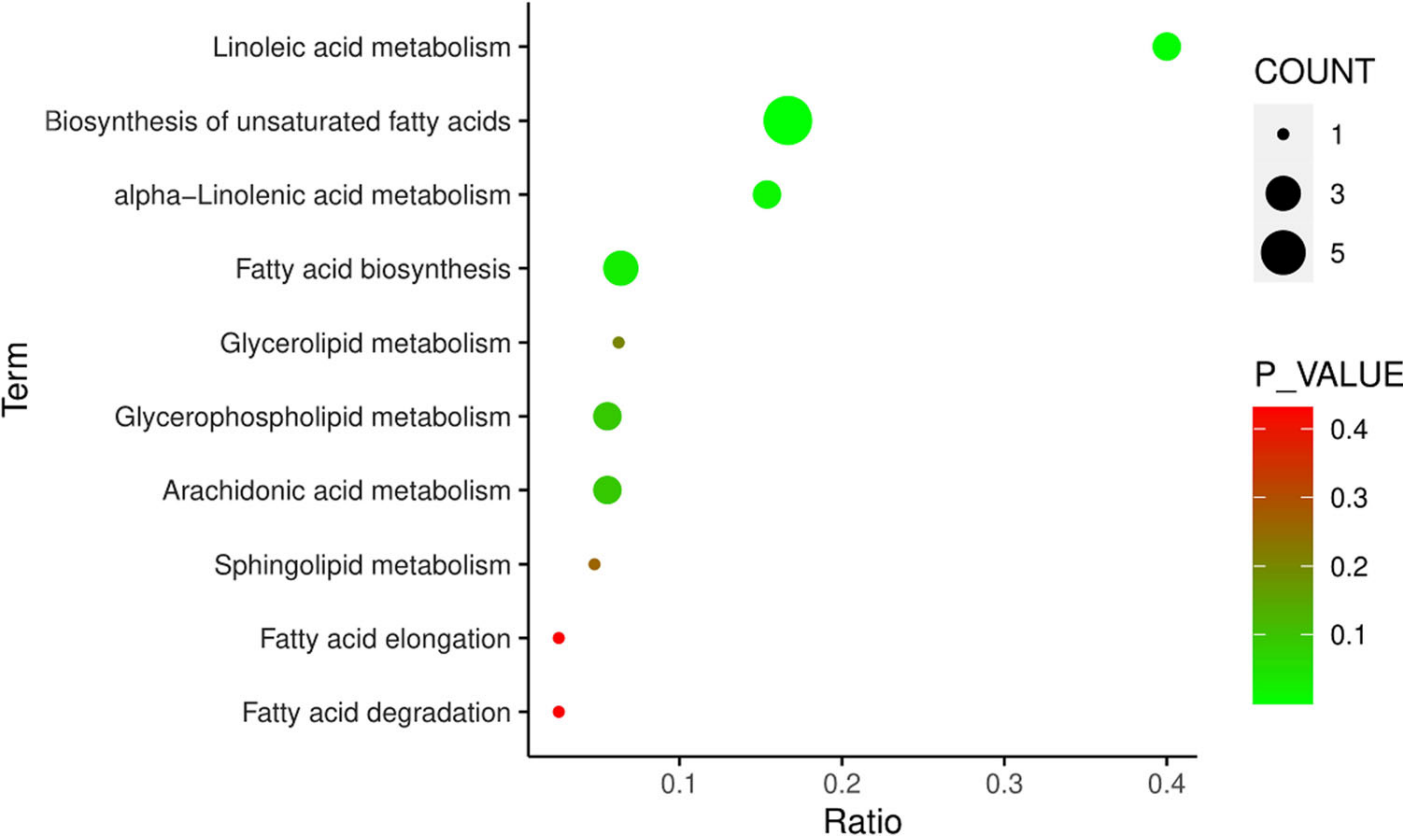
The 10 metabolic pathways were determined to be representative of IBS-D versus HC group by KEGG pathway analysis. The ratio (*x*-axis) shows the ratio of the different metabolites in the corresponding pathway, and the term (*y*-axis) shows the names of the ten pathways. The *P*-value is represented by the color of the circle, and the count of metabolites enriched in the pathway is indicated by the size of the circle

Moreover, we used KEGG pathway enrichment analysis to predict metabolic pathway based on these 97 identified differential metabolites. As shown in Table 1 and Fig. 3, of 10 pathways, 4 was determined to be most overrepresented in the IBS-D group compared to HC group: Biosynthesis of unsaturated fatty acids, Linoleic acid metabolism, alpha-Linolenic acid metabolism and Fatty acid biosynthesis.

**Table 1:** Summary of the 10 representative metabolic pathways in IBS-D group versus HC group

Pathway name	Hits/Total	*P*_value	− log(*P*)	FDR	Impact
Biosynthesis of unsaturated fatty acids	6/36	5.8214E−06	5.235	0.000489	0
Linoleic acid metabolism	2/5	0.001875	2.727	0.078749	1
alpha-Linolenic acid metabolism	2/13	0.013651	1.8648	0.38222	0.33333
Fatty acid biosynthesis	3/47	0.026861	1.5709	0.56409	0.01473
Glycerophospholipid metabolism	2/36	0.090621	1.0428	1	0.19895
Arachidonic acid metabolism	2/36	0.090621	1.0428	1	0.3135
Glycerolipid metabolism	1/16	0.20534	0.68752	1	0.01402
Sphingolipid metabolism	1/21	0.26079	0.58372	1	0
Fatty acid elongation	1/39	0.43136	0.36516	1	0
Fatty acid degradation	1/39	0.43136	0.36516	1	0

*Total* the number of all metabolites in the pathway, *Hits* the number of differentiated metabolites selected in the pathway

**Fig. 4:**
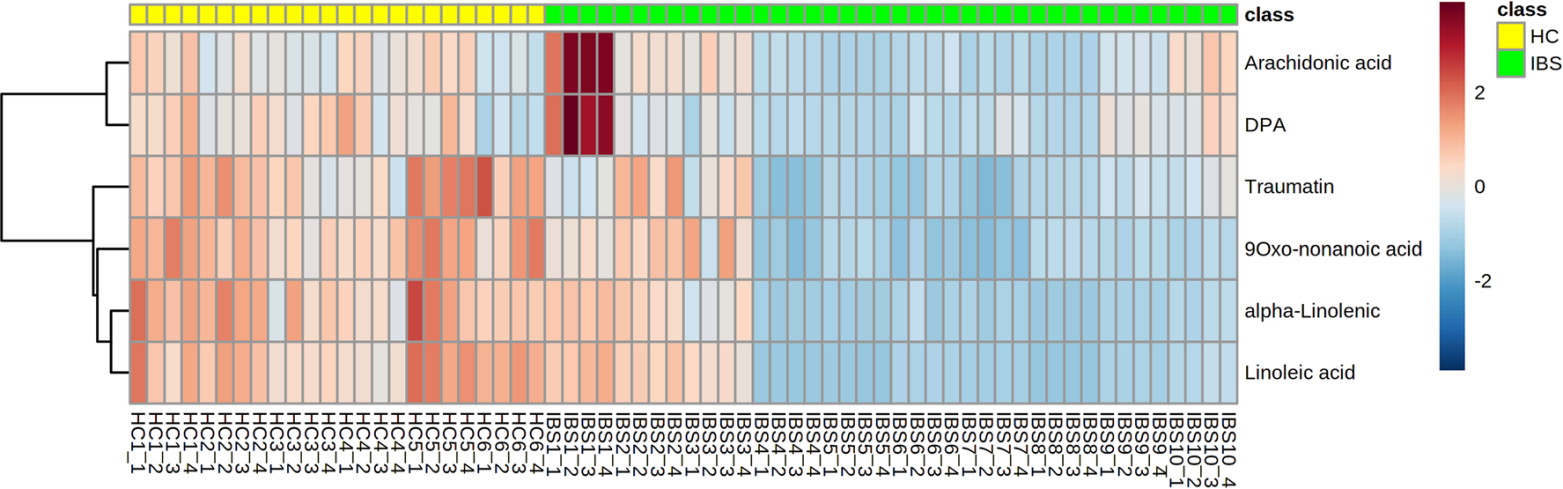
Heatmap of the six low-expressed differential metabolites for the IBS-D group samples versus the HC group samples. The colour-coding scale shows the relative intensity of each differential metabolite: red, high intensity; blue, low intensity; white, average intensity. The horizontal axis represents the samples and the vertical axis represents the differential metabolites. *n*(IBS-D) = 10, *n* (HC) = 6, every sample has 4 ROIs

**Fig. 5:**
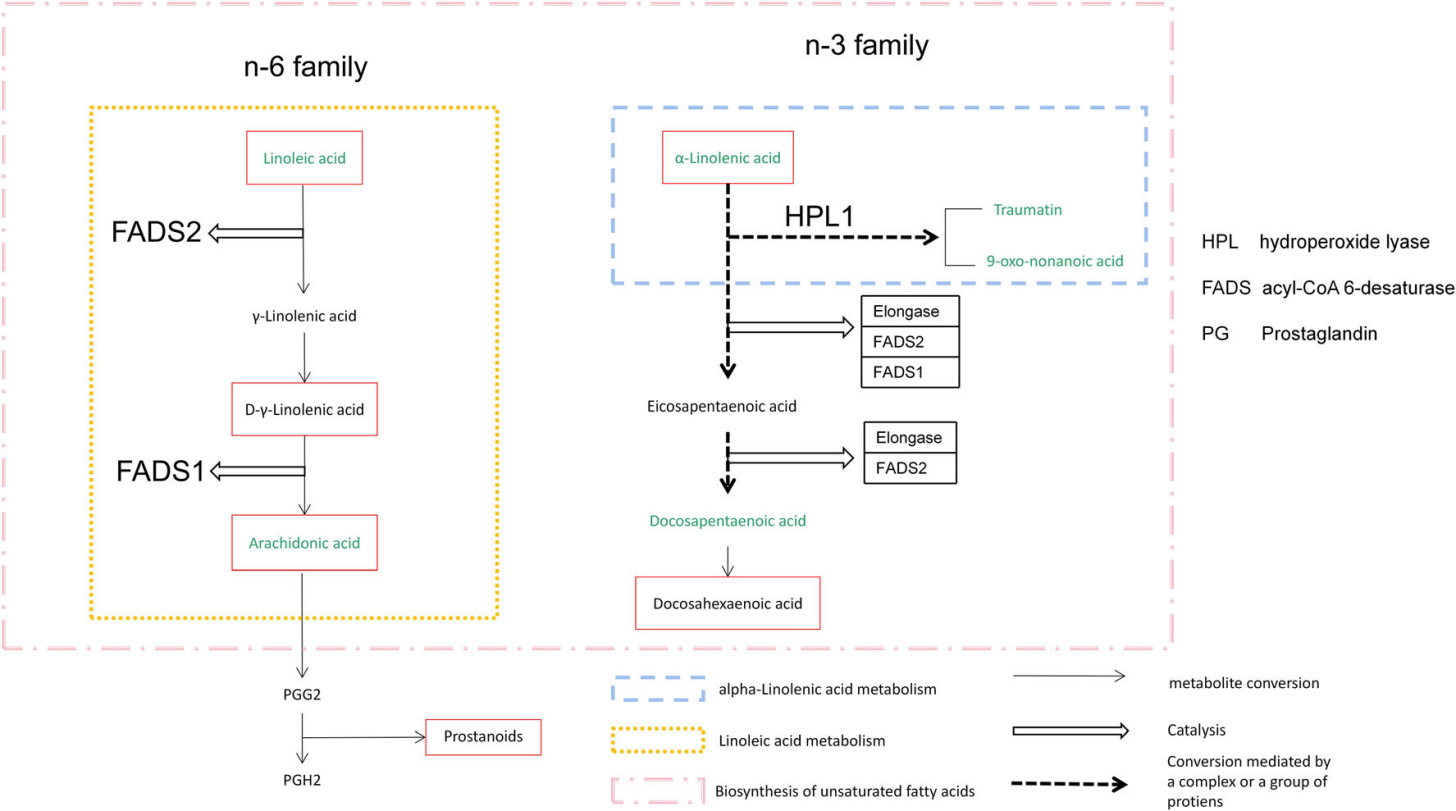
The diagram depicts the relationship between the six low-expressed metabolites (green font) in the mucosa layer of the IBS-D group and the metabolic pathways (dotted box) that may be involved in the occurrence of disease

**Fig. 6:**
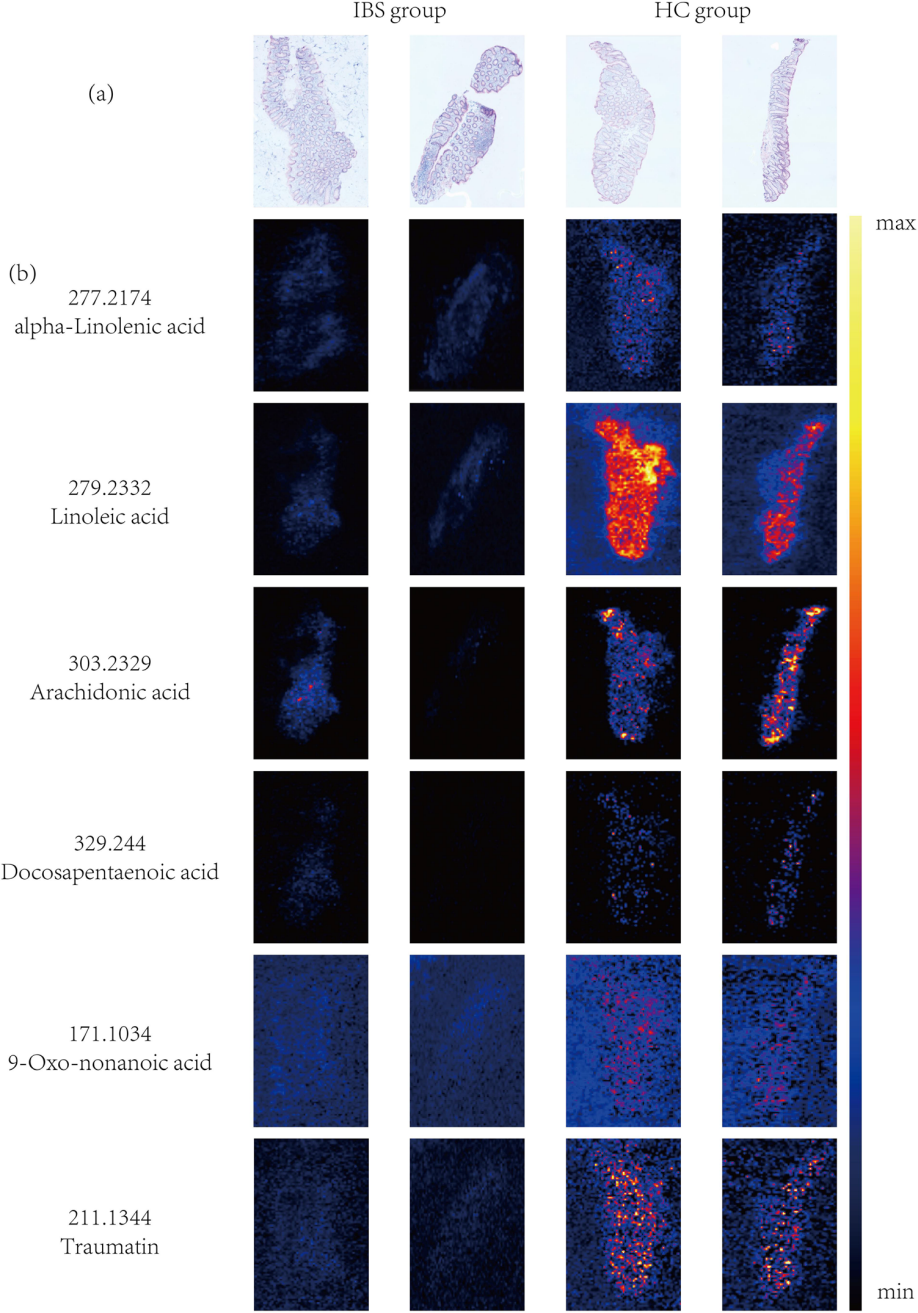
Visualization of spatial distribution and ion intensity of the differential metabolites for IBS-D. **a** The slices adjacent to the slices of DESI-MSI scanning were stained with H&E staining; **b** the distribution of six medium-chain and long-chain fatty acids in the IBS-D group and the HC group as determined by DESI-MSI

These differential metabolites and their metabolic pathways were further analyzed and discovered that, of 17 medium-chain and long-chain fatty acids, 6 fatty acids [Linoleic acid(LA), Arachidonic acid(AA), Alpha-Linolenic acid(ALA), Docosapentaenoic acid (DPA), 9-Oxo-nonanoic acid and Traumatin] were determined to be overrepresented metabolites and involved in down-regulated metabolic pathways in IBS-D patients (Figs. 4 and 5). LA, AA, ALA and DPA are all polyunsaturated fatty acids (PUFAs). DESI-MSI scanning can show us these fatty acids spatial distribution in colonic mucosa. We found that these fatty acids were distributed evenly in mucosa with higher abundances in HC group compared with IBS-D group (Fig. 6).

## Discussion

Data on the metabolic profile in IBS are urgently required, as the pathophysiology of this disease is largely unknown. DESI-MSI, as a new metabonomics technology, can be used to analyze metabolites and generate in situ imaging. DESI-MSI has not been reported on the metabolic studies in IBS patients. For the first time, we used DESI-MSI to identify colonic mucosa metabolic differences between IBS-D patients and healthy controls and characterize the spatial distribution of the screened metabolites. We found 97 metabolites in the colonic mucosa of IBS-D were significantly different compared with HC. Classifying these metabolites, 95 of them are lipids, except for 2 amino acids. Combine with KEGG pathway enrichment analysis showed that these metabolites were primarily involved in the pathways related to lipid metabolites, immune and inflammatory responses. Of the 95 lipid metabolites, 6 medium-chain and long-chain fatty acids show good performance in discriminating IBS-D from HC. DESI-MSI map comparing with pathological H&E section displayed that these metabolites were localized in the colonic mucous layer and confirmed the differences of these fatty acids between IBS-D and HC. These results suggested the potential biomarkers for differentiating IBS-D and HC.

In this study, we detected that 6 medium-chain and long-chain fatty acids, including AA, LA, ALA, DPA, traumatin and 9-Oxo-nonanoic acid, were abnormally down-regulated in the colonic mucosa metabolism of IBS-D. In normal physiological conditions, AA exists in the form of phospholipids on the cell membrane. When the cell membrane is stimulated, phospholipids are released from the cell membrane [13]. AA metabolism via cyclo-oxygenase (COX) and 5-lipoxygenase (LOX) leads to the formation of pro-inflammatory prostanoids and leukotrienes, respectively, which regulate immunity and inflammation [14, 15]. Several previous studies showed that the levels of AA in the plasma and stools of patients with IBS were increased [5, 16]. In this study, the content of AA in the colonic mucosa of IBS was significantly lower than that of healthy controls. It is speculated that the releasing AA from colonic mucosa into the blood may cause an increase in circulating levels. Moreover, a recent study reported that LA can achieve a bacteriostatic effect by inhibiting the biosynthesis of fatty acids in bacteria [17]. Some basic research suggested that ALA significantly reduced the expression of mRNA and protein of pro-inflammatory factors including TNF-α and IL-6, thereby alleviating intracellular oxidative stress [18, 19]. Nevertheless, DPA, Traumatin and 9-Oxo-nonanoic acid in our study have not been reported in previous metabolomic studies of IBS.

Further metabolic pathway analysis showed that the biosynthesis of unsaturated fatty acids, LA metabolism and ALA metabolism pathways in the colonic mucosa of IBS-D patients were all down-regulated, which was manifested as the decreased metabolites of LA, ALA, AA and DPA. In fact, these 4 metabolites are all PUFAs. PUFAs are divided into ω-6 family and ω-3 family according to the position of the first double bond. LA and ALA are the main representative precursor fatty acids of ω-6 family and ω-3 family, respectively. LA can also be converted into ALA through a series of reactions mediated by desaturase and carbon chain elongase [20]. Some basic medical studies have shown that various PUFAs and their metabolites can influence intestinal inflammatory processes, and inhibit the growth of various bacteria, fungi and viruses [17, 21]. Based on the physiological effects of PUFAs, it is speculated that abnormal biosynthesis of PUFAs may be involved in pathophysiological mechanisms in IBS-D.

DESI-MSI was used to map the spatial distribution of metabolites. From some studies on carcinoma DESI-MSI were applied and drew reliable conclusions [22]. In our study, four PUFAs (LA, ALA, AA and DPA) in both IBS-Ds and HCs were localized in the mucosal regions. The ion intensities of these four PUFAs in the mucosal regions of IBS-Ds were significantly lower than those of HCs, which was consistent with the results from the quantitative analysis in the previous results. A previous study provides evidence that links PUFA metabolites, TRPV4 activation and IBS-D. Its results suggested that PUFA metabolites produced by colonic tissues of IBS-D patients activate TRPV4 to induce hypersensitivity symptoms [23]. Bautzova et al. [24] demonstrated that PUFA 5-oxoETE (an ω-6 PUFA metabolite) was selectively increased in colonic biopsies from IBS patients with constipation by mass spectrometry. Local administration of 5-oxoETE induced somatic and visceral hypersensitivity without causing tissue inflammation, which suggested that this PUFA may mediate abdominal pain in patients with IBS [24]. Our results also suggested that as bioactive lipids, PUFA metabolites maybe utilised as biomarkers and therapeutic targets of IBS in the future. But the exact mechanism needs to be further studied and clarified.

Except for medium-chain and long-chain fatty acids, in our results, mucosa differential metabolites are almost lipids which is related to the metabolomics approach. DESI-MSI as a sensitive analytical tool for metabolomics, has its own limitations. It is necessary to carefully interpret the results, as large numbers of metabolites are included. The relevance of a single identified biomarker might not be high, but it could be that systematic up- or down-regulation in specific groups of molecules (such as PUFAs in the current study) indicates a biologically relevant metabolite type. Considering the limitations of the current study, one obvious weakness is the small sample size. But it is encouraging to find that IBS-D patients and healthy controls were well differentiated even with this limited number of subjects.

In conclusion, the current study is the first attempt to identify colonic mucosa metabolites of IBS using DESI-MSI technique. Our results suggested significant differences in the mucosa metabolic profile between IBS-D patients and healthy controls. In this study, IBS was particularly characterized by a down-regulation of medium-chain and long-chain fatty acids, such as several PUFAs. These lipid species have been associated with the modulation of pain sensitivity and inflammatory processes. Our data thus indicated that abnormal lipid metabolism might be involved in the pathophysiology of IBS and maybe also provide a new therapeutic target for IBS.

### Supplementary Information

Below is the link to the electronic supplementary material.

Supplementary file1 (PDF 215 KB)
