# Supplementary material for: Medium-chain and long-chain fatty acids are associated with diarrheal predominant irritable bowel syndrome revealed by DESI-MSI
Source: J Gastroenterol. 2023 Aug 14;58(11):1124–33. doi: 10.1007/s00535-023-02030-6 (PMC10590296; doi:10.1007/s00535-023-02030-6)
Supplement: Supplementary file 1 — Supplementary file1 (PDF 215 KB) [file 535_2023_2030_MOESM1_ESM.pdf]

Supplementary information

Article title: Medium-chain and long-chain fatty acids are associated with diarrheal predominant irritable bowel syndrome revealed by DESI-MSI

Journal names: Journal of Gastroenterology

Author names: Yanli Zhang • Huiting Zhu • Shiyu Du • Huifen Wang • Hui Li • Miao Wang• Bing Shao

The affiliation and address of the author:

1.Department of Gastroenterology, China-Japan Friendship Hospital, Beijing 100029,China

2.Beijing Key laboratory of Diagnostic and Traceability Technologies for Food Poisoning,Beijing Centers for Disease Control and Preventative Medical Research, Beijing 100013,China

3.Department of Gastroenterology, First Hospital of Qinhuangdao, Qinhuangdao 066000,Hebei Province,China

Corresponding Author: Bing Shao

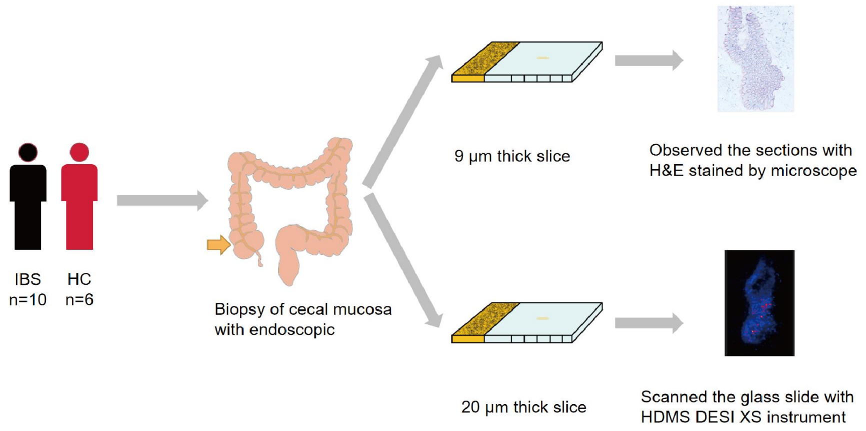

Supplementary Fig.1 Flow chart

Supplementary Table 1 Clinical characteristics of patients with IBS-D and Healthy control(HC) groups

	IBS-D group (n=10)	HC group (n=6)	*P* value
Age(years)	34.2±6.8	38.3±10.6	0.39
Gender (Male: Female)	7: 3	4: 2	1.00
BMI (kg/m^2)	24.8±4.3	24.3±2.2	0.81
IBS-SSS	257.7±80.7	NA	NA

Supplementary Table 2 The differences in metabolites between the IBS-D group and HC group

Measured m/z	Theoretical m/z	Delta	Molecular formula	Adduct	Description	Trend compared with controls
327.2221	327.2177	-0.0044	$C_{18}H_{32}O_5$	M-H	(9R,10S,12Z)-9,10-Dihydroxy-8-oxo-12-octadecenoic acid	DOWN
660.4061	660.4001	-0.0060	$C_{32}H_{64}NO_8P$	M+K	1,2-Dilauroyl-sn-glycero-3-phosphocholine	UP
239.0605	239.0673	0.0068	$C_9H_{10}N_2O_3$	M+FA-H	4-Aminohippuric acid	DOWN
171.1034	171.1027	-0.0007	$C_9H_{16}O_3$	M-H	9-Oxo-nonanoic acid	DOWN
277.2174	277.2173	-0.0001	$C_{18}H_{30}O_2$	M-H	alpha-Linolenic acid	DOWN
311.2953	311.2956	0.0003	$C_{20}H_{40}O_2$	M-H	Arachidic acid	DOWN
303.2329	303.233	0.0001	$C_{20}H_{32}O_2$	M-H	Arachidonic acid	DOWN
187.0983	187.0976	-0.0007	$C_9H_{16}O_4$	M-H	Azelaic acid	DOWN
634.5397	634.5405	0.0008	$C_{39}H_{68}O_5$	M+NH4	DG(16:0/20:4(5Z,8Z,11Z,14Z)/0:0)	UP
638.5682	638.5718	0.0036	$C_{39}H_{72}O_5$	M+NH4	DG(18:1(9Z)/18:1(9Z)/0:0)	UP
741.5475	741.5434	-0.0041	$C_{44}H_{78}O_6$	M+K	DG(20:4(5Z,7E,11Z,14Z)-OH(9)/0:0/i-21:0)	DOWN
704.5847	704.5829	-0.0018	$C_{43}H_{74}O_6$	M+NH4	DG(20:5(6E,8Z,11Z,14Z,17Z)-OH(5)/0:0/i-20:0)	UP
718.6296	718.6349	0.0053	$C_{45}H_{80}O_5$	M+NH4	DG(20:2n6/0:0/22:2n6)	UP
725.5563	725.5562	-0.0001	$C_{42}H_{76}O_9$	M+H	DG(TXB2/0:0/i-19:0)	DOWN
146.0467	146.046	-0.0007	$C_5H_9NO_4$	M-H	DL-Glutamate	DOWN
329.244	329.2486	0.0046	$C_{22}H_{34}O_2$	M-H	Docosapentaenoic acid (22n-3)	DOWN
199.1707	199.1704	-0.0003	$C_{12}H_{24}O_2$	M-H	Dodecanoic acid	DOWN
182.0099	182.0129	0.0030	$C_4H_9NO_5S$	M-H	Homocysteic acid	UP
279.2332	279.233	-0.0002	$C_{18}H_{32}O_2$	M-H	Linoleic acid	DOWN
227.2019	227.2017	-0.0002	$C_{14}H_{28}O_2$	M-H	Myristic acid	DOWN
281.2484	281.2486	0.0002	$C_{18}H_{34}O_2$	M-H	Oleic acid	DOWN
678.4064	678.3977	-0.0087	$C_{33}H_{57}O_{11}P$	M+NH4	PA(20:4(7E,9E,11Z,13E)-3OH(5S,6R,15S)/10:0)	UP
805.575	805.5753	0.0003	$C_{47}H_{83}O_8P$	M-H	PA(24:1(15Z)/20:4(8Z,11Z,14Z,17Z))	DOWN
803.5613	803.5596	-0.0017	$C_{47}H_{81}O_8P$	M-H	PA(24:1(15Z)/20:5(5Z,8Z,11Z,14Z,17Z))	DOWN
867.6815	867.6813	-0.0002	$C_{49}H_{97}O_8P$	M+Na	PA(i-24:0/i-22:0)	DOWN
682.4235	682.4289	0.0054	$C_{33}H_{61}O_{11}P$	M+NH4	PA(PGF1alpha/10:0)	UP
255.2331	255.233	-0.0001	$C_{16}H_{32}O_2$	M-H	Palmitic acid	DOWN

253.2175	253.2173	-0.0002	$C_{16}H_{30}O_2$	M-H	Palmitoleic acid	DOWN
761.588	761.5803	-0.0077	$C_{41}H_{78}NO_8P$	M+NH4	PC(15:0/18:2(9Z,12Z))	UP
734.5732	734.5695	-0.0037	$C_{40}H_{80}NO_8P$	M+H	PC(16:0/16:0)	UP
760.5844	760.5851	0.0007	$C_{42}H_{82}NO_8P$	M+H	PC(18:1(11Z)/16:0)	UP
758.5704	758.5695	-0.0009	$C_{42}H_{80}NO_8P$	M+H	PC(18:1(11Z)/16:1(9Z))	UP
786.6002	786.6008	0.0006	$C_{44}H_{84}NO_8P$	M+H	PC(18:1(11Z)/18:1(9Z))	UP
794.5427	794.5341	-0.0086	$C_{44}H_{78}NO_9P$	M-H	PC(18:1(9Z)-O(12,13)/18:3(9Z,12Z,15Z))	DOWN
804.5724	804.576	0.0036	$C_{42}H_{82}NO_8P$	M+FA-H	PC(20:1(11Z)/14:0)	DOWN
850.5566	850.5568	0.0002	$C_{45}H_{82}NO_{10}P$	M+Na	PC(20:4(8Z,11Z,14Z,17Z)-2OH(5S,6R)/17:0)	DOWN
864.5147	864.515	0.0003	$C_{48}H_{76}NO_9P$	M+Na	PC(20:5(6E,8Z,11Z,14Z,17Z)-OH(5)/20:5(5Z,8Z,11Z,14Z,17Z))	DOWN
828.5722	828.576	0.0038	$C_{44}H_{82}NO_8P$	M+FA-H	PC(22:2(13Z,16Z)/14:1(9Z))	DOWN
848.563	848.5566	-0.0064	$C_{46}H_{84}NO_8P$	M+K	PC(20:4(5Z,8Z,11Z,14Z)/18:0)	DOWN
870.6865	870.6946	0.0081	$C_{50}H_{96}NO_8P$	M+H	PC(24:1(15Z)/18:1(11Z))	DOWN
920.7202	920.7103	-0.0099	$C_{54}H_{98}NO_8P$	M+H	PC(24:1(15Z)/22:4(7Z,10Z,13Z,16Z))	DOWN
854.7069	854.6997	-0.0072	$C_{50}H_{96}NO_7P$	M+H	PC(24:1(15Z)/P-18:1(11Z))	DOWN
867.702	867.695	-0.0070	$C_{50}H_{92}NO_7P$	M+NH4	PC(O-22:1(13Z)/20:4(8Z,11Z,14Z,17Z))	DOWN
895.7255	895.7263	0.0008	$C_{52}H_{96}NO_7P$	M+NH4	PC(O-22:2(13Z,16Z)/22:3(10Z,13Z,16Z))	DOWN
893.7163	893.7106	-0.0057	$C_{52}H_{94}NO_7P$	M+NH4	PC(O-22:3(10Z,13Z,16Z)/22:3(10Z,13Z,16Z))	DOWN
674.4244	674.4163	-0.0081	$C_{33}H_{66}NO_8P$	M+K	PE(14:0/14:0)	UP
744.5525	744.5549	0.0024	$C_{41}H_{80}NO_8P$	M-H	PE(14:0/22:1(13Z))	DOWN
672.4077	672.4001	-0.0076	$C_{33}H_{64}NO_8P$	M+K	PE(14:1(9Z)/14:0)	UP
700.4222	700.432	0.0098	$C_{35}H_{68}NO_8P$	M+K	PE(16:1(9Z)/14:0)	UP
748.5179	748.5134	-0.0045	$C_{38}H_{74}NO_8P$	M+FA-H	PE(18:1(9Z)/15:0)	DOWN
726.4501	726.4476	-0.0025	$C_{37}H_{70}NO_8P$	M+K	PE(18:2(9Z,12Z)/14:0)	UP
722.5082	722.513	0.0048	$C_{41}H_{74}NO_7P$	M-H	PE(18:3(6Z,9Z,12Z)/P-18:1(11Z))	DOWN
848.5462	848.5436	-0.0026	$C_{47}H_{78}NO_{10}P$	M+H	PE(20:3(8Z,11Z,14Z)-2OH(5,6)/22:5(7Z,10Z,13Z,16Z,19Z))	DOWN
742.538	742.5392	0.0012	$C_{41}H_{78}NO_8P$	M-H	PE(14:0/22:2(13Z,16Z))	DOWN
822.5681	822.5654	-0.0027	$C_{45}H_{80}NO_7P$	M+FA-H	PE(22:5(7Z,10Z,13Z,16Z,19Z)/P-18:0)	UP
750.5439	750.5443	0.0004	$C_{43}H_{78}NO_7P$	M-H	PE(P-18:0/20:4(5Z,8Z,11Z,14Z))	DOWN
241.2175	241.2173	-0.0002	$C_{15}H_{30}O_2$	M-H	Pentadecanoic acid	DOWN
861.5449	861.5499	0.0050	$C_{45}H_{83}O_{13}P$	M-H	PI(20:2(11Z,14Z)/16:0)	DOWN
887.5576	887.5655	0.0079	$C_{47}H_{85}O_{13}P$	M-H	PI(20:3(5Z,8Z,11Z)/18:0)	DOWN
885.5477	885.5499	0.0022	$C_{47}H_{83}O_{13}P$	M-H	PI(20:4(5Z,8Z,11Z,14Z)/18:0)	DOWN
802.5578	802.5604	0.0026	$C_{43}H_{82}NO_{10}P$	M-H	PS(22:1(13Z)/15:0)	DOWN
862.5499	862.5451	-0.0048	$C_{44}H_{82}NO_{13}P$	M-H	PS(PGF1alpha/18:0)	DOWN
788.5968	788.5912	-0.0056	$C_{42}H_{79}N_2O_8P$	M+NH4	SM(d17:1/20:3(8Z,11Z,14Z)-2OH(5,6))	UP
703.5724	703.5748	0.0024	$C_{39}H_{79}N_2O_6P$	M+H	SM(d18:0/16:1(9Z))	UP
367.3578	367.3582	0.0004	$C_{24}H_{48}O_2$	M-H	Tetracosanoic acid	DOWN
881.7421	881.7359	-0.0062	$C_{55}H_{102}O_5$	M+K	TG(14:0/20:3(5Z,8Z,11Z)/O-18:0)	DOWN
856.7434	856.7389	-0.0045	$C_{54}H_{94}O_6$	M+NH4	TG(14:0/22:5(4Z,7Z,10Z,13Z,16Z)/15:0)	DOWN
852.7082	852.7075	-0.0007	$C_{54}H_{90}O_6$	M+NH4	TG(14:1(9Z)/15:0/22:6(4Z,7Z,10Z,13Z,16Z,19Z))	DOWN
825.6886	825.6942	0.0056	$C_{51}H_{94}O_6$	M+Na	TG(14:1(9Z)/16:0/18:1(9Z))	DOWN
903.7215	903.7202	-0.0013	$C_{57}H_{100}O_5$	M+K	TG(14:1(9Z)/22:5(4Z,7Z,10Z,13Z,16Z)/O-18:0)	DOWN
882.7585	882.7545	-0.0040	$C_{56}H_{96}O_6$	M+NH4	TG(15:0/16:0/22:6(4Z,7Z,10Z,13Z,16Z,19Z))	DOWN

906.7613	906.7545	-0.0068	$C_{58}H_{96}O_6$	M+NH4	TG(15:0/20:3n6/20:5(5Z,8Z,11Z,14Z,17Z))	DOWN
878.7258	878.7232	-0.0026	$C_{56}H_{92}O_6$	M+NH4	TG(15:0/20:4(5Z,8Z,11Z,14Z)/18:4(6Z,9Z,12Z,15Z))	DOWN
908.7729	908.7702	-0.0027	$C_{58}H_{98}O_6$	M+NH4	TG(15:0/20:4(8Z,11Z,14Z,17Z)/20:3(5Z,8Z,11Z))	DOWN
851.7404	851.7463	0.0059	$C_{54}H_{100}O_5$	M+Na	TG(15:0/O-18:0/18:3(6Z,9Z,12Z))	DOWN
827.709	827.7099	0.0009	$C_{51}H_{96}O_6$	M+Na	TG(16:0/16:0/16:1(9Z))	DOWN
854.7277	854.7232	-0.0045	$C_{54}H_{92}O_6$	M+NH4	TG(15:0/20:5(5Z,8Z,11Z,14Z,17Z)/16:1(9Z))	DOWN
851.7085	851.7099	0.0014	$C_{53}H_{96}O_6$	M+Na	TG(16:0/16:0/18:3(9Z,12Z,15Z))	DOWN
877.7253	877.7256	0.0003	$C_{55}H_{98}O_6$	M+Na	TG(16:0/16:0/20:4(5Z,8Z,11Z,14Z))	DOWN
871.7157	871.7151	-0.0006	$C_{53}H_{100}O_6$	M+K	TG(16:0/16:1(9Z)/18:0)	DOWN
881.7543	881.7569	0.0026	$C_{55}H_{102}O_6$	M+Na	TG(16:0/16:1(9Z)/20:1(11Z))	DOWN
891.6812	891.6838	0.0026	$C_{55}H_{96}O_6$	M+K	TG(16:0/16:1(9Z)/20:4(5Z,8Z,11Z,14Z))	DOWN
905.7557	905.7569	0.0012	$C_{57}H_{102}O_6$	M+Na	TG(16:0/16:0/22:4(7Z,10Z,13Z,16Z))	DOWN
879.741	879.7412	0.0002	$C_{55}H_{100}O_6$	M+Na	TG(16:0/18:1(9Z)/18:2(9Z,12Z))	DOWN
919.7151	919.7151	0.0000	$C_{57}H_{100}O_6$	M+K	TG(16:0/18:1(9Z)/20:4(5Z,8Z,11Z,14Z))	DOWN
893.7005	893.6995	-0.0010	$C_{55}H_{98}O_6$	M+K	TG(16:0/18:2(9Z,12Z)/18:2(9Z,12Z))	DOWN
907.7692	907.7725	0.0033	$C_{57}H_{104}O_6$	M+Na	TG(16:0/18:2(9Z,12Z)/20:1(11Z))	DOWN
901.721	901.7256	0.0046	$C_{57}H_{98}O_6$	M+Na	TG(16:0/18:2(9Z,12Z)/20:4(5Z,8Z,11Z,14Z))	DOWN
907.7521	907.7515	-0.0006	$C_{57}H_{104}O_5$	M+K	TG(16:0/20:4(5Z,8Z,11Z,14Z)/O-18:0)	DOWN
895.716	895.7151	-0.0009	$C_{55}H_{100}O_6$	M+K	TG(16:1(9Z)/18:1(9Z)/18:1(9Z))	DOWN
917.7015	917.6995	-0.0020	$C_{57}H_{98}O_6$	M+K	TG(16:1(9Z)/18:1(9Z)/20:4(5Z,8Z,11Z,14Z))	DOWN
853.7223	853.7256	0.0033	$C_{55}H_{96}O_6$	M+H	TG(16:1(9Z)/18:2(9Z,12Z)/18:2(9Z,12Z))	DOWN
923.746	923.7464	0.0004	$C_{57}H_{104}O_6$	M+K	TG(16:1(9Z)/18:2(9Z,12Z)/20:0)	DOWN
921.73	921.7308	0.0008	$C_{57}H_{102}O_6$	M+K	TG(16:1(9Z)/18:2(9Z,12Z)/20:1(11Z))	DOWN
869.7018	869.6995	-0.0023	$C_{53}H_{98}O_6$	M+K	TG(18:0/16:1(9Z)/16:1(9Z))	DOWN
878.7562	878.7596	0.0034	$C_{57}H_{96}O_5$	M+NH4	TG(18:4(6Z,9Z,12Z,15Z)/18:4(6Z,9Z,12Z,15Z)/O-18:0)	DOWN
211.1344	211.134	-0.0004	$C_{12}H_{20}O_3$	M-H	Traumatin	DOWN

Supplementary table 3 List of lipid name abbreviations

Lipid name abbreviation	Lpid name
GLP	Glycerophospholipid
FA	Fatty acid
SM	Sphingomyelin
TG	Triglyceride
DG	Diglyceride
PC	Phosphatidylcholine
PE	Phosphatidylethanolamine
PS	Phosphatidylserines
PI	Phosphatidylinositols
PA	Phosphatidic acids
PG	Phosphatidylglycerols
